# High-Speed Videography Reveals How Honeybees Can Turn a Spatial Concept Learning Task Into a Simple Discrimination Task by Stereotyped Flight Movements and Sequential Inspection of Pattern Elements

**DOI:** 10.3389/fpsyg.2018.01347

**Published:** 2018-08-03

**Authors:** Marie Guiraud, Mark Roper, Lars Chittka

**Affiliations:** ^1^School of Biological and Chemical Sciences, Queen Mary University of London, London, United Kingdom; ^2^Drone Development Lab, Ben Thorns Ltd, Colchester, United Kingdom; ^3^Wissenschaftskolleg, Institute of Advanced Study, Berlin, Germany

**Keywords:** active vision, *Apis mellifera*, cognition, feature detection, local features, video tracking, visual learning

## Abstract

Honey bees display remarkable visual learning abilities, providing insights regarding visual information processing in a miniature brain. It was discovered that bees can solve a task that is generally viewed as spatial concept learning in primates, specifically the concept of “above” and “below.” In these works, two pairs of visual stimuli were shown in the two arms of a Y-maze. Each arm displayed a “referent” shape (e.g., a cross, or a horizontal line) and a second geometric shape that appeared either above or below the referent. Bees learning the “concept of aboveness” had to choose the arm of the Y-maze in which a shape–*any shape*–occurred above the referent, while those learning the “concept of belowness” had to pick the arm in which there was an arbitrary item beneath the referent. Here, we explore the sequential decision-making process that allows bees to solve this task by analyzing their flight trajectories inside the Y-maze. Over 368 h of high-speed video footage of the bees' choice strategies were analyzed in detail. In our experiments, many bees failed the task, and, with the possible exception of a single forager, bees as a group failed to reach significance in picking the correct arm from the decision chamber of the maze. Of those bees that succeeded in choosing correctly, most required a close-up inspection of the targets. These bees typically employed a close-up scan of only the bottom part of the pattern before taking the decision of landing on a feeder. When rejecting incorrect feeders, they repeatedly scanned the pattern features, but were still, on average, faster at completing the task than the non-leaners. This shows that solving a concept learning task could actually be mediated by turning it into a more manageable discrimination task by some animals, although one individual in this study appeared to have gained the ability (by the end of the training) to solve the task in a manner predicted by concept learning.

## Introduction

Concept learning is often viewed as a key ingredient of what makes humans uniquely intelligent, since it appears to involve a number of mental abstractions (e.g., equivalence, area, volume, and numerosity) (Piaget and Inhelder, 1956; Marcus et al., 1999), as well as sentence constructions and mathematical operations (Edward et al., 1992; Chen et al., 2004; Christie et al., 2016). Yet, in the last 50 years, concept learning has been a recurrent theme when exploring animal cognition (Savage-Rumbaugh et al., 1980; Savage-Rumbaugh, 1984; Akhtar and Tomasello, 1997; Zayan and Vauclair, 1998; Depy et al., 1999; Penn et al., 2008; Shettleworth, 2010). Scientists have discovered concept learning in various animal taxa, for example the learning of sameness and difference concepts in the pigeon (Zentall and Hogan, 1974), in ducklings (Martinho and Kacelnik, 2016), monkeys (Wright et al., 1984), the honeybee (Giurfa et al., 2001), and one study comparing two species of monkeys and pigeons (Wright and Katz, 2006); other studies focused on oddity and non-oddity in monkeys (Moon and Harlow, 1955), pigeons (Lombardi et al., 1984; Lombardi, 2008), rats (Taniuchi et al., 2017), sea lions (Hille et al., 2006), dogs (Gadzichowski et al., 2016), and honeybees (Muszynski and Couvillon, 2015); the concept of symmetry/asymmetry in honeybees (Giurfa et al., 1996). Spatial concepts such as aboveness and belowness have been explored in a number of vertebrates (Zentall and Hogan, 1974; Depy et al., 1999; Spinozzi et al., 2004), and also the honeybee (Avarguès-Weber et al., 2011, 2012). However, the majority of studies have focused on whether or not the subject could solve a given task, not on how the animals actually solved them. Similar tasks might be solved by profoundly different mechanisms and behavioral strategies in different animal species.

**Figure 1:**
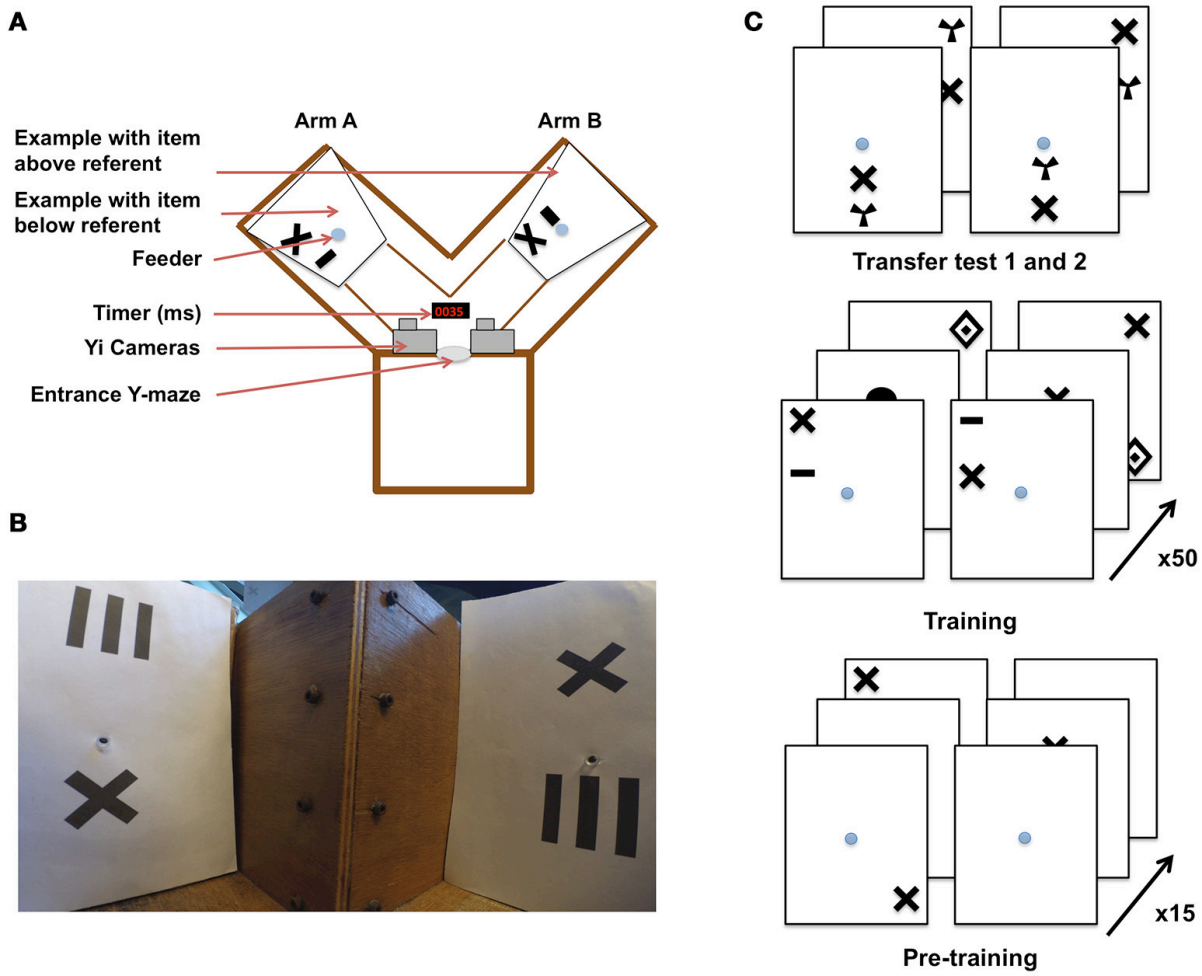
Y-maze setup and training procedure for honeybees in an “aboveness–belowness” spatial learning task. **(A)** Schematic representation of the experimental setup. The Y-maze presents, on one side, the “above stimulus” with one of the five geometrical shapes above the referent cross, and on the other side the “below stimulus” where the same geometrical shape is below the cross. The center of the sheet contains the feeder where the bee has to crawl into a tunnel to get the reward. A timer is present to synchronize both cameras installed above the setup. **(B)** View of the Y-maze setup, taking the “bee perspective” from the decision chamber; the cross is, again, the referent. The “above” configuration is shown on the left, and the “below” configuration on the right. **(C)** Example of the conditioning and testing procedure. From bottom to top: bees were exposed to 15 pre-training bouts where a cross on a white sheet was rewarded (50% sugar solution) while the plain white sheet was associated with saturated quinine solution. Training consisted of 50 trials with “above configuration” in one arm and “below configuration” in the other one. The transfer tests were not rewarded. Half of the bees were rewarded on the “target above referent” relation whereas the other half was rewarded on the “target below referent” relation.

In a typical protocol to explore potential concept learning animals, Avarguès-Weber et al. (2011) tested honeybees in a series of binary choices in a Y-maze flight arena to assess whether bees could master the conceptual spatial relationships of “above” and “below.” The experimental paradigm consisted of a pair of stimuli, each with a variable geometric target shape located above or below a shape (e.g., a black cross) that acted as referent point (Figure 1). One of the arms of the Y-maze flight arena presented the target above the referent and the other presented the same target but below the referent. The bees had to learn that either the “above” or “below” pattern configuration was associated with reward (sucrose solution provided in the center of the stimulus wall), and the other pattern lead to a punishment (quinine solution). After fifty training bouts, bees were subjected to an unrewarded transfer test using novel target shapes to determine if they had learnt the concept of “aboveness” or “belowness.” The results indicated that positive transfer occurred (Avarguès-Weber et al., 2011). These trials did not, however, show *how* bees solved the problem, and therefore a number of alternative hypotheses might potentially explain these results. Indeed, depending on how bees approach the task during training, they may evaluate their options and learn differently. Bees trained to an “aboveness” task could simply fly to referent (the invariant component of the display) and verify that the ventral visual field is empty (without examining the item above the referent). The reverse solution could be applied in a “belowness” task. Bees could approach a stimulus scanning only the top (or the bottom) shape and learn that they should either expect the referent in that position, or anything other than the referent (depending on whether they are learning “above” or “below”). Avarguès-Weber et al. (2011) proposed that bees evaluate the whole compound stimulus, using the relative position of the stimuli shapes to determine their “above” or “below” relationship, and then choose accordingly. It is also conceivable that different individuals use different strategies when faced with the same task, or indeed that the same individuals use different strategies in different phases of their training.

We tested these hypotheses to understand the bees' strategies in solving such tasks by replicating the original honeybee “above and below” experiments (Avarguès-Weber et al., 2011), but with the addition of high-speed cameras to record the flight paths during every training and transfer test trial. We aimed to determine *how* variations in the bees' behavior toward the stimuli during the training impacted their learning abilities, and subsequent transfer test performances. We evaluated which shapes or stimuli regions the bees inspected (including the order of elemental observations and repetitions) time spent in each activity, as well as performances during and after training.

## Materials and methods

### Setting and material

Experiments were conducted over three consecutive summers (2015–2017). Honeybees (*Apis mellifera*) from three colonies were allowed to collect 20% sucrose solution (w/w) from a gravity feeder located either 20 m or 2 m from the hives depending on the year. This type of feeder is shown in (Frisch (1965) his Figure 18)–it allows several dozen bees to feed simultaneously and commute between the hive and the feeder. This ensures that a good number of motivated foragers are typically available near the training setup. An individual bee is then tempted away from this communal feeding station by offering it a reward that is higher in quality than that of the gravity feeder. In our experiments, we offered a cotton bud, soaked with 50% sucrose solution (w/w) to a bee that had just landed near the gravity feeder. Once the bee walked on the cotton bud, and began feeding, she was slowly transferred by the experimenter to one of the feeding tubes within the apparatus. A small colored dot was applied to the bee's dorsal abdomen using colored Posca marking pens (Uni-Ball, Japan), while she was feeding. Upon the return from the hive, the bee was typically found back at the gravity feeder or near the setup. This procedure was repeated until the bee learnt to fly directly to the feeding tubes at the end of the Y-maze arms (the bee was put in either the left or right arm in a pseudo-random sequence, no more than twice in the same arm and usually needed a repetition of two to three of these operations before the task can be initiated). This method allowed us to limit the number of bees near the apparatus. Additionally, any unmarked bees were removed from the experimental area. Only one bee was trained at a time within the Y-maze, and we followed the original experimental protocol (Avarguès-Weber et al., 2011), albeit with some modifications. The Y-maze (see Figure 1) consisted of an entrance hole that led to a central decision chamber, from which two arms extended. Each arm measured 40 × 20 × 20 cm (L × H × W). Within each arm, a moveable rear wall (20 × 20 cm) was placed 15 cm from the decision chamber, providing support for the stimuli and feeder tubes. Unlike the experiments by Avarguès-Weber et al. (2011), no Perspex transparent cover was placed on the top of the Y-maze flight arena; this was to allow for an unobstructed and undistorted view while taking high-speed video recordings. Two Yi (Xiaomi Inc. China) sport cameras were positioned side-by-side 10 cm above the entrance of the Y-maze. Their field of view was adjusted such that they looked down into the arena at ~60° from horizontal, establishing in both cameras a wide-angle view of both arms. Each Yi camera was configured to record at 120 fps (frames per second) at a resolution of 720 p (1,280 × 720 pixels). Once the bee entered the arena, both cameras were started, such that there was an individual video file per camera, per trial. Filming of a trial began when the honey bee entered the flight arena and continued until the bee entered the rewarding feeding tube.

Each stimulus was composed of black patterns on a 20 × 24 cm (W × H) white UV-reflecting paper, printed using a high-resolution laser printer. The patterns were disposed of after a single use, to prevent odors being deposited by the bees and being subsequently used as an olfactory cue during learning. Another modification of the setup by Avarguès-Weber et al. (2011) was that we had to modify the feeding stations. In the earlier study, this was a tube that protruded into the arena, and was filled with sucrose solution from the side of the arena. We performed a pilot study, collecting high speed video footage of two bees and found that bees made brief antennal touches to the feeders during fly-bys, allowing them to assess whether they contained sucrose solution prior to the decision to land (Supplementary Video 1) (Such antennal contacts are so brief that they are practically undetectable to the naked eye or with conventional video footage). To prevent bees from such contacts, our visual stimuli were combined with a centrally located feeding tube (1 × 0.5 cm) that led to 50% sucrose solution (w/w) (see Figure 1 for protocol). This was implemented to prevent sucrose solution being deposited on the entrance of the feeding tube during refilling, thereby forcing the honeybees to crawl into the tube (or at least land and put the head in the tube, see Supplementary Videos 2–5) to determine if it contained a reward. These feeding tubes were cleaned between trials, again to prevent odor cues being used in subsequent trials. Blank brown cardboard cover-plates 20 × 20 × 0.5 cm were placed in front of each of the two stimuli to prevent a bee from seeing the patterns or accessing the feeding tubes before a trial had begun. Two pairs of achromatic patterns were presented during each trial.

### Phase 1–Pre-training

For Phase 1 pre-training, the pair of stimuli consisted of blank white paper for one arm, and in the other white paper with a black cross (4 × 4 cm), which was later used as the “referent” in training (Figure 1). Each individual bee was first trained using an absolute conditioning protocol (Giurfa et al., 1999) in the Y-maze with the rewarding pattern presented in each arm in a pseudo-random sequence. In this, we followed the published protocol of Avarguès-Weber et al. (2011), where such a pseudorandom choice of the Y-maze arm was also reported. The rewarding stimulus was always a black cross randomly positioned on the white background. The other arm of the maze contained a fresh blank white sheet of paper (unpaired stimulus) with the feeding tube providing an aversive quinine solution. The bee's first choice (e.g., the bee touching and entering the feeder) was recorded and acquisition curves produced by calculating the frequency of correct choices per block of five trials. After 15 training trials, a discrimination test was introduced. In this test, two patterns were used; one consisted of the familiar cross, and the other contained one of five alternative shapes (to be used as targets in later training: concentric diamonds (5 × 6 cm), a small horizontal bar (1 × 3 cm), a vertical grating (5 × 5 cm), a filled circle (3 cm in diameter), or a radial three-sectored pattern (4 × 4 cm) (Avarguès-Weber et al., 2011). Neither pattern was rewarding, with both feeding tubes leading to 30 μl of water. The bee entered as normal but was given 45 s to explore the new configuration. The number of visits to each feeding tube was recorded.

### Phase 2–Main training

In the main training phase, bees that completed phase 1 pre-training were either subjected to an “above,” or a “below” differential conditioning protocol (see Figure 1). Each stimulus contained a pair of shapes. One was the same cross as used during pre-training, and which was now the “referent,” being present in all stimuli. The other shape was a geometric “target” shape which could be either concentric diamonds, a small horizontal bar, a vertical grating, a filled circle, or a radial three-sectored pattern (Avarguès-Weber et al., 2011). These target shapes were horizontally aligned with the cross (either above or below it) and the pair of shapes (“referent” cross and “target”) positioned randomly on the paper (centered, top-left, bottom-right, etc.). Two stimuli were presented in each trial, one pair in each of the Y-maze arms. Both stimuli contained the referent cross shape and another shape selected from the four available target shapes (excluding the shape used for that bee's phase 1 discrimination test). When the bee was assigned to the “above” group (Group A) she had to learn that the rewarding pattern would be the stimulus where the target would appear above the referent cross, and this arrangement would be associated with *ad libitum* 50% sucrose solution (w/w). The other stimulus (CS– or negative conditioned stimulus) presented the target below the cross and its feeding tube led to saturated quinine solution (Group B, “below” bees were trained with the reciprocal stimulus being aversive). If the bee chose the CS–, it tasted the quinine solution, and was allowed to continue flying within the flight arena inspecting the patterns until it discovered the rewarding feeder. The CS+ and CS– stimuli were presented in a pseudo-random sequence (never more than two consecutive trials on the same arm, see Figure 1) to prevent the bees, as far as possible, from learning a side preference. After feeding, the bee would depart for the hive, and return approximately every 3–10 min. This interval allowed for the next pairs of stimuli to be inserted into the Y-maze. The bees were trained for 50 trials. The first feeder choice was recorded upon the bee entering the maze after returning from the hive. Acquisition curves were produced by calculating the frequency of correct choices per block of 10 trials. Following the last acquisition trial, non-rewarded tests were performed with novel stimuli (utilizing the 5th geometric shape excluded from the training trials). During the tests, both the first feeder choice and the cumulative contacts with the feeders were counted for 45 s. The choice proportion for each of the two test stimuli was then calculated. Each test was performed twice, interchanging the sides of the stimuli to control for side preferences. Three rewarding trials using the training stimuli were conducted between the tests to ensure that foraging motivation did not decay owing to non-rewarded test experiences.

### Video analysis

The videos for each of the 50 training trials, for each bee, were replayed on a computer monitor in slow motion (1/8th of the regular speed) so that the particular flight trajectories of the bee could be observed and annotated. We analyzed 46h of raw footage (368 in slow motion) of videos to create the dataset. The honeybees typically displayed three types of flight characteristics during a trial: (a) direct flights: in these instances, the bees would enter the flight arena and fly directly to one or other of the feeding tubes (Supplementary Video 2). These flights would take less than a second until the bee had landed on the feeding tube, (b) scanning behavior: here the bees would either briefly fly toward one of the pattern shapes (0.5–2.0 s, brief inspection; Supplementary Video 3) or scan the shape with slow horizontal movements, repeated several times, with a typical duration between 1 and 15 s (Supplementary Video 4); (c) repetitive scans after a wrong decision: bees would successively scan feeder, top shape and bottom shape a number of times before changing arm (Supplementary Video 5).

**Figure 2:**
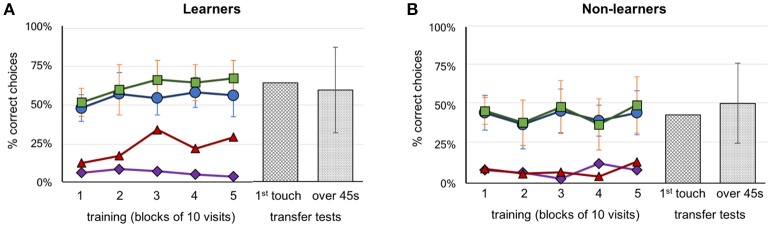
Performance of bees during training and transfer tests. **(A)** Group A (above configuration, *n* = 4) and Group B (below configuration, *n* = 7) learner bees. **(B)** Group A (above configuration, *n* = 5) and Group B (below configuration, *n* = 3) non-learner bees. Five blocks of 10 trials are represented with the percentage of correct choices. Green squares: number of correct feeders, blue circles: selection of correct Y-maze arm first, red triangles: abandoned incorrect arm for a correct feeder (the higher the better), purple diamonds: abandoned correct arm for an incorrect feeder (the lower the better). Transfer test results, hatched bars: percentage of correct first touches, dotted bars: percentage of accumulative touches on correct feeder. Error bars show standard deviation.

Our video analysis focused on recording the following types of behaviors: side preference (upon entering the Y-maze, whether the bee displayed a consistent preference for the left or right arm of the apparatus when first selecting an arm during a trial); correct arm choice (if the bee initially selected the arm that contained the correctly configured pattern or CS+ arm), direct flights (if the bee flew directly to a feeder without scanning the patterns, recorded for both CS+ and CS– arms), and all scanning points (which component of the pattern the bee visited (bottom shape, top shape, and center (feeder). This included both scanning behavior and the less common brief inspections of shapes. A bee was designated as a “learner,” if during the last 20 trials of complete training, it achieved an average of at least 60% correct choices *and* had at least 70% correct choices in one block of these two blocks of 10 trials. Otherwise it was classified as a non-learner bee (see Figure 2). Performance of balanced groups during acquisition was compared using Kruskal–Wallis *H* tests, and statistics within groups and trial blocks were tested using Mann–Whitney *U* tests, as well as tests against chance. All statistics were calculated using Python programming language.

## Results

### Training and tests performance

Each bee took between 8 and 16 h to complete the training and testing phases. Thirty-seven bees that commenced training failed to complete the full protocol (either the bee did not return to the experiment after a trial, or poor weather conditions interrupted the bees' foraging). In total, 21 honey bees were trained. Two were excluded because they were mistakenly exposed to three or more rewarding patterns on the same Y-maze arm (A6 and B7, see Supplementary Figures 1, 2 for individual data). Of the remaining 19 bees, 9 bees were trained on the “above” protocol (Group A bees) and 10 bees on the “below” protocol (Group B bees). Seven of the ten Group B bees were successful at learning their task (correct stimulus having target shapes below the crosses). In contrast, only four individual bees from Group A learnt to identify the patterns with the target shapes above the crosses (Figure 2). Thus, in total eight bees (42%) failed to learn the task in our experiments; this contrasts with previous experiments (Avarguès-Weber et al., 2011) where all bees were reported to solve the task. Unless otherwise indicated, groups from the “above protocol” and “below protocol” were pooled, as there were no significant differences between them (these non-significant results are given in Supplementary Tables 1–7). Figure 2 shows a summary of these results grouped into the learner and non-learner bees (Individual results: Supplementary Figures 1, 2). Overall, the bees we had classified as learners exhibited training performances which improved over time [Kruskal–Wallis *H*_(2)_ = 10.5; *df* = 10; *P* = 0.03] while non-learners did not [Kruskal–Wallis *H*_(2)_ = 3.7; *df* = 7; *P* = 0.454]. Moreover, the learner group selected the correct feeder 61% of the time, with bees averaging 66.4% over the last 10 trials; these bees performed significantly better than chance over each of the last three blocks of 10 trials (Mann–Whitney *U* for learners' training: *df* = 10; trials 21–30: *U* = 16.5, *P* = 0.004; trials 31–40: *U* = 16.5, *P* = 0.004 and trials 41–50: *U* = 16.5, *P* = 0.004; Supplementary Table 1). The non-learner group of bees, on the other hand, selected the correct feeder 44% of the time, with bees averaging 48.8% over the last 10 trials. These bees did not perform significantly better than chance over each of the last three blocks of 10 trials (Mann–Whitney *U* for learners training: *df* = 7; trials 21–30: *U* = 20, *P* = 0.226; trials 31–40: *U* = 16, *P* = 0.103 and trials 41–50: *U* = 28, *P* = 0.711; Supplementary Table 1).

During the transfer tests, bees were presented with stimuli using a novel target shape, above or below the familiar referent crosses. The learner group exhibited a preference for the correct stimulus during transfer tests with 63.6% (Mann–Whitney *U* for correct choices during test above chance for learners: *U* = 38.5, *df* = 10; *P* = 0.045; Supplementary Table 2). Similar results were seen for the average percentage of correct touches over the 45 s tests (58.8%; Mann–Whitney *U* test–choices for correct stimulus above chance for learners: *U* = 22.0, *df* = 10; *P* = 0.00; Supplementary Table 2). Although statistical analysis shows significance for correct choices, individual bees differed widely in the investigation of unrewarded stimuli, and also in terms of performances according to the sequence of the tests (first or second unrewarded test; see Supplementary Figures 1, 2). The non-learner group of bees did not perform any better than chance, achieving just 43.8% (Mann–Whitney test *U* = 28.0; *df* = 7; *P* = 0.335; Supplementary Table 2) for first choice during tests, and 51.2% (Mann–Whitney test *U* = 16.0, *df* = 7; *P* = 0.173; Supplementary Table 2) correct percentage of accumulative touches over 45 s, respectively (Figure 2).

In earlier works it was reported that bees were able to solve the task by using the spatial configuration of the elements of the stimuli (e.g., the target in relation to the referent) when viewing both patterns from the decision chamber, and choosing a Y-maze arm accordingly (Avarguès-Weber et al., 2011, 2012). In our study, we found that 13 of the 19 bees that completed training exhibited a strong side preference when entering the setup (a choice of left or right arm of ≥70%). Unsurprisingly perhaps, given the widespread nature of side biases, bees of the learner group did not choose the correct arm of the Y-maze significantly more than chance (Mann–Whitney *U* = 33.0, *df* = 11; *P* = 0.077; Supplementary Table 3). However, given this significance level we cannot reject with certainty the possibility that these bees initiated their decision making process in the decision chamber, and tended to do so correctly. Indeed, a single individual managed 90% correct choices *from the decision chamber* in the final 10 visits of training. This individual had already had above average performance throughout training (when making decisions close up to the target area) and appeared to switch strategies near the end of training so that choices were now initiated in the decision chamber (Supplementary Figure 2, bee: B8).

However, learner bees as a group failed to reach significance in choosing the correct Y-maze arm. We then evaluated the decision making process once bees had entered the arms of the Y maze. We first asked if the initial (accidental or *via* side bias) selection of the correct arm led to the choice of the correct feeder. Bees in the learner group selected the rewarding feeder more than 94.6% of the time after initially having entered the correct Y-maze arm, leading to no difference between the number of times they chose the correct arm, and the number of times they chose the correct feeder after choosing the correct arm (Mann–Whitney *U* = 42, *df* = 10, *P* = 0.238, no difference, thus high similarity; Supplementary Table 3). This behavior was also observed in the non-learner groups (Mann–Whitney *U* = 23.0, *df* = 7, *P* = 0.373; Supplementary Table 3) (Figure 2). However, the learner group of bees showed an ability to revert an incorrect first choice of a Y-maze arm during training by inspecting the stimulus but subsequently choosing to go to the other arm and select the feeder there. When the individuals of the learner groups entered an incorrect arm, they abandoned the arm a total of 48 out of the 252 incorrect choices (19%), and an average of 28.3% of such occurrences during the last 10 trials. This significantly differed from the non-learner group, which only left the wrong Y-maze arm 14 out of 228 wrong arm visits (6.1%) (Mann–Whitney *U*, difference between learner and non-learner groups *U* = 2.0, *df* = 18, *P* < 0.001; Supplementary Table 4).

### Spatial conceptual learning or discrimination task?

Having shown that a subset of our bees (learners from both the “above” and “below” groups) solved their respective tasks, we used the high-speed video recordings captured during each trial to analyze the sequential choices of both learner and non-learner group of bees during training. Avarguès-Weber et al. (2011) suggested that bees could use the spatial relationship between the two shapes present in the stimulus to solve the task. In this condition, bees would need to either make their decisions at some distance from the patterns (i.e., from the decision chamber), or by sequentially inspecting the two shapes within a pattern before choosing one of the feeders.

However, upon entering a Y-maze arm, bees did not fly directly to a feeder but typically spent time scanning the stimulus in the selected Y-maze arm. Interestingly, in all conditions below, no significant differences were found between learners and non-learners, thus both groups were pooled (Supplementary Tables 5–7). For analysis, three options were considered: bees could go directly to the feeder (Supplementary Video 2), scan the bottom shape (Supplementary Video 4), or the top shape. In all cases, chance represents 33.3% (50 trials and three options).

**Figure 3:**
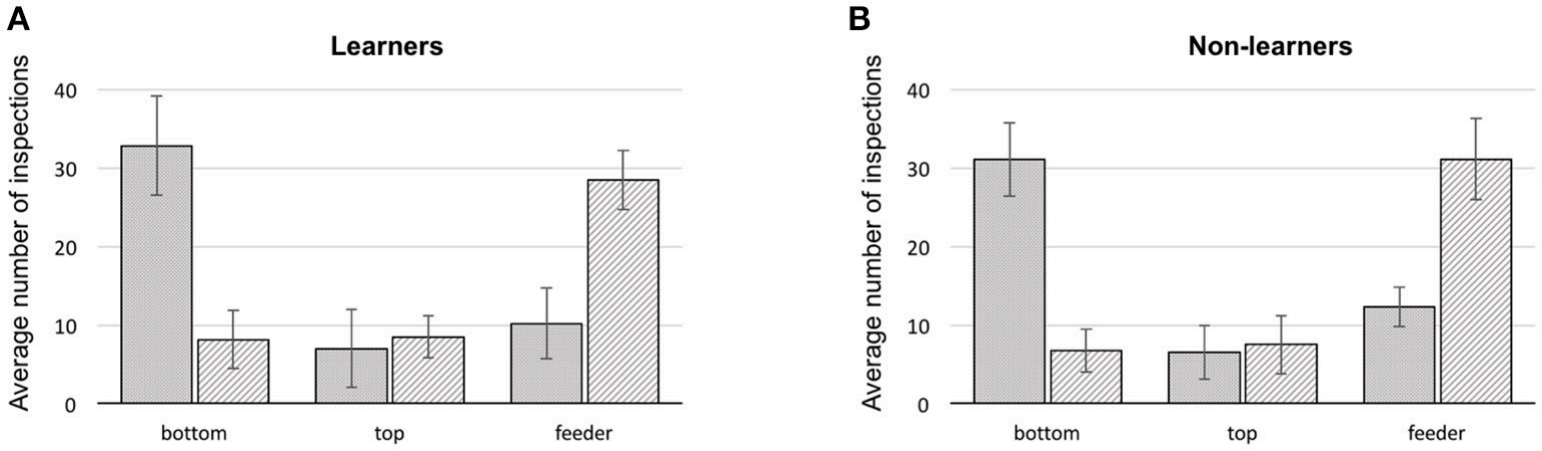
Summary of first and second scanned locations for bees during training. **(A)** learners, **(B)** non-learners. Solid: average number of 1st scans at a stimulus location (error bars: standard deviation). Hatched: average number of 2nd scans at a location (error bars: standard deviation), bottom: lowest shape presented on a stimulus, top: upper most shape, feeder: either a scan in front of, or landing on the feeder.

In all bees, the first item scanned was the bottom shape of the stimulus, in 64.2% of the cases (bottom choice vs. chance (33.3%) Mann–Whitney *U* = 0.0, *df* = 18, *P* = 0.0; Supplementary Table 5). The remaining 35.8% were split between feeder and top item. Collectively, in just 22.2% of flights did bees fly directly to a feeder. The majority of the direct flights to a rewarding feeder were by the learners (65.3%), constituting 10.7% of their trials. Similarly, 9.8% of learner bee flights were directly to the wrong feeders. Flying directly toward the top shape of the stimulus occurred in only 13.7% of total trials (Figure 3).

We additionally analyzed how each group of bees made use of targets and referents (see Supplementary Figures 3, 4 and Supplementary Data Sheets 1, 2). However, this analysis only confirmed that bees from all groups have a strong preference for scanning the bottom item first (independent of whether it was a target or cross shape). Bees did not usually choose a feeder as their first location approached (as one might expect if the decision had been arrived at in the decision chamber of the Y-maze). Even if the arrangement of items in a stimulus was analyzed only by close-up scanning to solve the task, the logical following choice would be to scan the top item after the initial inspection of the bottom item. Yet, of the three options (top, bottom item and feeder) the second inspection point for any bee would often be one of the two feeders, in 58.1% of the cases (difference from a chance expectation of 33.3%–Mann–Whitney *U* = 0.0, *df* = 18, *P* = 0.0; Supplementary Table 6) although learners appear to choose feeders as second scanning item less (56% on average) than bees of the non-learner group (64.1% on average).

### Differences between learners and non-learners

To explore the causes of differences in performance between learners and non-learners, we evaluated the number of items scanned by the bees and the intervals between entering the setup, scanning items, and selecting a feeder.

Over the entire 50 training bouts, the average cumulative number of scanning behaviors by each bee was 375.3 (±60.8) (minimum: 265; maximum: 523). Learners tended to display fewer inspections overall (362.5 ± 59.3) than non-learners (392.9 ± 62.4) but there was pronounced individual variation and therefore no significant difference between groups (learners vs. non-learners: Mann–Whitney *U* = 167.0, *df* = 18, *P* = 0.39; Supplementary Table 7). Interestingly, more inspections were made by learners (98.1 ± 23.8) than non-learners bees (77.6 ± 54.6) before making a correct choice (Mann–Whitney *U* = 20, *df* = 19, *P* = 0.026; Supplementary Table 7). Learners approached and scanned another item than the feeding tube in 96% of the cases before making a correct choice vs. 45% for non-learners, which, in in two-third of the cases would be the lower item of the stimulus. Moreover, non-learners displayed slightly more inspection behavior (315.3 ± 90.3 items inspected) than learners (264.5 ± 63.3) when making an incorrect decision but this difference is not significant (Mann–Whitney *U* = 28, *df* = 18, *P* = 0.1; Supplementary Table 7). For learners and non-learners, the number of scanning behavior increased strongly after an incorrect choice (by a factor of 2.7 for learners and 4.1 for non-learners). When making an incorrect choice, after first probing the quinine solution, the bee will typically exhibit a repetitive sequence of scanning behaviors of the feeder, the top and bottom shape of the stimulus a multiple times before departing to the opposite arm of the Y-maze). The number of items scanned was 9.8 on average and ranged from 1 to 47. Conversely, a bee making a correct decision will typically feed and leave the setup without any subsequent scanning of the stimulus features.

These results indicate that the learner group of honeybees tend to be more efficient. They need to scan only one item before making a correct decision (96% of the time), and they need to scan fewer items after making an incorrect choice (1.5 times less than non-learners).

## Discussion

Our findings confirm the ability of bees to solve the “above and below” visual learning task. The authors of the original study on spatial concept learning in bees (Avarguès-Weber et al., 2011) managed to train all their bees to solve the task, whereas approximately half of our bees failed. The relatively poorer performance of bees in our study may be a result of colony differences or local weather, wind and lighting conditions (Raine and Chittka, 2008; Arnold and Chittka, 2012; Ravi et al., 2016). They might also result from subtle differences in experimental procedures; for example, to facilitate video-tracking, we did not use a lid on the flight arena during experiments, and we took special care to prevent any odor cues or pheromones being deposited on the apparatus by changing the stimuli and washing all tubes before each new trial in the training phase, as well as before tests. In the study by Avarguès-Weber et al. (2011), fresh (unscented) stimuli were used only in tests (not during training), which shows that in their study, bees *were* able to solve the tasks without the availability of scent, but some of the quantitative differences in learning performance of bees in the two studies might result from the scent cues available during training in Avarguès-Weber et al. (2011).

Individual differences in problem solving abilities are well-documented in insects (Chittka et al., 2003), especially with difficult tasks (Alem et al., 2016), and it may thus be unsurprising that some individuals failed the task. To explore the question of how the more capable individuals solved the task, it is therefore meaningless to evaluate performance of the entire group, in the same way as one could not study the mnemonic strategies used by people with extraordinary memory capacity by taking a population average that includes all people that lack such capacities. In such cases, one must establish a criterion by which to distinguish the learners from the non-learners. Because of the relatively poor overall performance of bees in our study (compared to that reported by Avarguès-Weber et al., 2011), we chose a relatively lenient criterion (at least 60% overall correct choices during the last 20 trials of learning *and* at least 70% correct choices during at least one of the two last blocks of 10 trials). Using this criterion, 11 of the 19 bees in our study managed to learn their respective tasks within the 50 training trials and were, on average, able to transfer to the novel stimuli, showing a higher proportion of both first touches and accumulative touches on the stimuli with the correct spatial arrangements (Figure 2).

For the question of whether the task was learnt in a manner consistent with concept learning, it is crucial to evaluate whether bees surveyed the arrangement of items in a pair from a distance, and whether the predicted arm of the Y-maze was chosen accordingly. In our study, bees as a group failed to select the Y-maze arm containing the correct stimuli from the flight arena decision chamber. However, our results for the learner group of bees (that relatively narrowly miss significance at the 5% level) cannot strictly rule out the possibility that, as suggested by Avarguès-Weber et al. (2011), these bees might initiate the decision-making process from a distance, and indeed one individual bee in our study achieved 90% correct choices (from the decision chamber) at the end of training. In our experiments, however, the analysis of the high-speed video footage reveals that much of the decision making process happens when bees were close to the target walls in the Y-maze, when stimuli are scanned close-up, and that the task can be solved without the formal need for concept learning, by simply scanning the bottom item and making decisions accordingly.

The primary aim of this study was to investigate *how* bees solve the “aboveness” and “belowness” tasks. We aimed to determine what strategies and mechanisms the bees might employ during the learning process, and we therefore video-recorded every single training trial and test. It is generally assumed that the “above and below” task requires a subject to form a conceptual rule to solve the problem, and especially to transfer this ability to novel, correctly configured visual stimuli. However, other explanations might be possible. Three hypotheses were stated in our introduction: bees could recognize the invariant part of each stimulus (the referent), approach it and then depending on whether there is an item (*any* item) below the referent, decide if it is the correct pattern (simply by noting that the visual field below the referent is empty for “aboveness” learners, or that the visual field above the referent is empty for “belowness” learners). Bees could approach a stimulus scanning only the top (or the bottom) shape and learn that they should either expect the referent in that position, or anything other than the referent (depending on whether they are learning “above” or “below”). Finally, in line with the notion of concept learning, bees could evaluate a whole compound stimulus, using the relative position of the stimuli shapes to determine their “above” or “below” relationship (e.g., scanning both items successively, or viewing the entire arrangement from a distance), and then choose accordingly.

Analysis of the first scanning points showed that the bees were not initially scanning just the referents (crosses), but mostly the lowest shapes presented on the stimuli of the chosen arms (in line with hypothesis 2). This was true for all bees irrespective of their training protocol, or indeed of whether they were successful at the task or not. In approximately two-thirds of cases, the first item scanned by the bees was the lowest presented shape on a stimulus. Furthermore, the second scanning behavior was most often performed in front of a feeder. Therefore, bees did not appear to employ a strategy based on finding the referent (cross in our study) or the spatial relationship between the referent and the other geometrical shape (target). Instead, they used a visual discrimination approach, flying first toward the lower shape and evaluate if it is the referent or not; they do not have to attend to, or indeed learn, anything about the targets. After initially choosing an arm of the Y-maze randomly or according to a side bias, bees trained to the “above” task simply have to decide if the chosen arm contains the referent cross as the lower shape—if yes, they are in the correct arm and can proceed to the feeder. If not, they must have chosen the incorrect arm. Bees trained in the “below” task, finding the referent cross as the lower item, know that they are in the wrong arm; finding “anything but the referent” as the lower item means that they are in the correct arm and can feed. This interpretation is in line with a previous study showing that bees will only learn the lower half of a pattern if this is sufficient to solve a given discrimination task (Giurfa et al., 1999).

## Conclusion

Our analysis of the honey bees' flight characteristics showed that the “above and below” problem can be solved using a clever sequential inspection of items rather than, strictly speaking, a spatial concept. By simply flying into a random arm of the Y-maze, or flying into an arm based on a side preference, the task can be solved by inspecting the lower of two shapes in a pair in any arm of the Y-maze, the bee can decide whether it has arrived in the correct arm of the Y-maze or not. It may be tempting to assume that this strategy of solving a seemingly complex learning task might be more suitable for a miniature nervous system such as a bee's, but it will be interesting to explore whether the same strategy may actually be employed by animals with much larger brains when solving similar tasks, such as pigeons (Kirkpatrick-Steger and Wasserman, 1996), chimpanzees (Hopkins and Morris, 1989), baboons (Depy et al., 1999), and capuchins (Spinozzi et al., 2004), or indeed, may be used by humans if they are not verbally instructed how to solve the task. Other studies have reported further forms of concept learning in bees (Giurfa et al., 2001; Avarguès-Weber and Giurfa, 2013; Howard et al., 2018) and in these cases, too, it will be useful to explore the sequential decision making process to see if bees find behavioral strategies to simplify the task or whether concept formation is the most plausible explanation. Finally, it is also possible that bees (and other animals) switch strategies during more prolonged training, so that they might initially learn tasks by close-up inspections of visual targets such as those reported here, and later switch to a more cognitive strategy that allows solving the puzzle from a distance and with higher speed.

Our exploration of the strategy by which bees solve a seemingly complex cognitive task raises questions on the very nature of complexity in comparative cognition. All too often, researchers in that field classify as “advanced cognition” what appears to be clever behavior by casual inspection—but without an analysis of either the behavioral strategy used by animals or a quantification of the computational requirements, or indeed an exploration of the neural networks underpinning the observed behavior (Chittka et al., 2012). Recent computational models of information processing in the bee brain reveal that various forms of “higher order” cognition can emerge as a property of relatively simple neural circuits (Peng and Chittka, 2017; Roper et al., 2017). On the other hand, “simple” associative learning can result in such wide-ranging changes in neural circuitry that these can be detected by sampling just tiny fractions of a principal association region of the bee, the mushroom bodies (Li et al., 2017). These observations, and our analysis of behavior strategies reported here, that the traditional ranking of cognitive operations from simple, non-associative learning through associative learning to apparently more complex such as rule and “abstract concept” learning may have to be fundamentally revised, and may require more than just asking whether or not animals are clever.

## Ethics statement

This study does not involve human subjects. The 1986 EU Directive 86/609/EEC on the protection of animals used for scientific purposes defines “Animal” as any live non-human vertebrate. Therefore, this does not apply to experimental work on insects.

## Author contributions

MR and LC conceived the study. MG and MR elaborated the project, methods, and collected the data. MG analyzed video footage. MR participated in the data analysis. MG, MR, and LC wrote the manuscript.

### Conflict of interest statement

The authors declare that the research was conducted in the absence of any commercial or financial relationships that could be construed as a potential conflict of interest.
