# Supplementary material for: High-Speed Videography Reveals How Honeybees Can Turn a Spatial Concept Learning Task Into a Simple Discrimination Task by Stereotyped Flight Movements and Sequential Inspection of Pattern Elements
Source: Front Psychol. 2018 Aug 3;9:1347. doi: 10.3389/fpsyg.2018.01347 (PMC6086205; doi:10.3389/fpsyg.2018.01347)
Supplement: Supplementary file 8 [file Presentation_1.pdf]

Supplementary Material

High-speed videography reveals how honeybees can turn a spatial concept learning task into a simple discrimination task by stereotyped flight movements and sequential inspection of pattern elements

Marie Guiraud[1]†, Mark Roper[1,2]†, Lars Chittka[1,3]*

1 School of Biological and Chemical Sciences, Queen Mary University of London

2 Drone Development Lab, Ben Thorns Ltd, Colchester, UK

3 Wissenschaftskolleg / Institute of Advanced Study, Wallotstrasse 19, 14193 Berlin, Germany

† Authors contributed equally to the work

*Corresponding author: Lars Chittka l.chittka@qmul.ac.uk

Supplementary Video 1. Video footage of a close-up inspection of a feeder by a honey bee. We initially replicated the feeder design used by Avarguès-Weber et al. (2011; Proc Biol Sci 278, 898-905) with a feeding tube that protruded from the plane of stimulus presentation. The video shows an approaching forager first in real time (up to 3s of the video) and then in a slow-motion repeat (speed 1/8th; 3-16s of the video). The slow-motion reveals that bees can sample the solution contained in the feeder while still in flight, by extremely brief antennal contacts. This allows bees to make decisions prior to landing, and so we had to build a modified feeder design for the experiments reported in this study. In our design, bees had to crawl inside a feeding tube that was refilled with sucrose solution from the side pointing away from the interior of the Y-maze

Supplementary Video 2. A direct flight into the correct arm of the maze and to the feeder by a bee. The animal had been trained to the "above" task and therefore had to select the arm in which an item was displayed above the reference cross. The subject flew directly into the correct (left) arm, did not inspect any of the cues present in the pattern, and flew straight to the feeder.

Supplementary Video 3. A bee performing a brief inspection ("glance") of the lower item of the stimulus before landing on the feeder. Such inspections usually last less than one second. The bee had been trained to the "above" task, and hence the Y-maze arm chosen (left) is this correct arm since it presents an item above the referent cross.

Supplementary Video 4. Subject performing a thorough scan of the lower item for more than three seconds before flying to the feeder. The bee had been trained to the "above" task and therefore chose the left arm of the maze (in which an item was displayed above the referent cross) correctly.

Supplementary Video 5. A bee making an incorrect choice and displaying typical behavior following an error. This bee was trained in the “above” task and should therefore have chosen the left arm of the Y-maze, in which the target was displayed above the referent cross. Instead, she flew into the right arm, and after a brief inspection of the bottom pattern, she tasted the bitter quinine solution. She then briefly scanned the feeder, both items of the stimulus, as well as empty areas of the white stimulus sheet, before flying to the opposite arm of the Y-maze, where she is rewarded with sucrose solution.

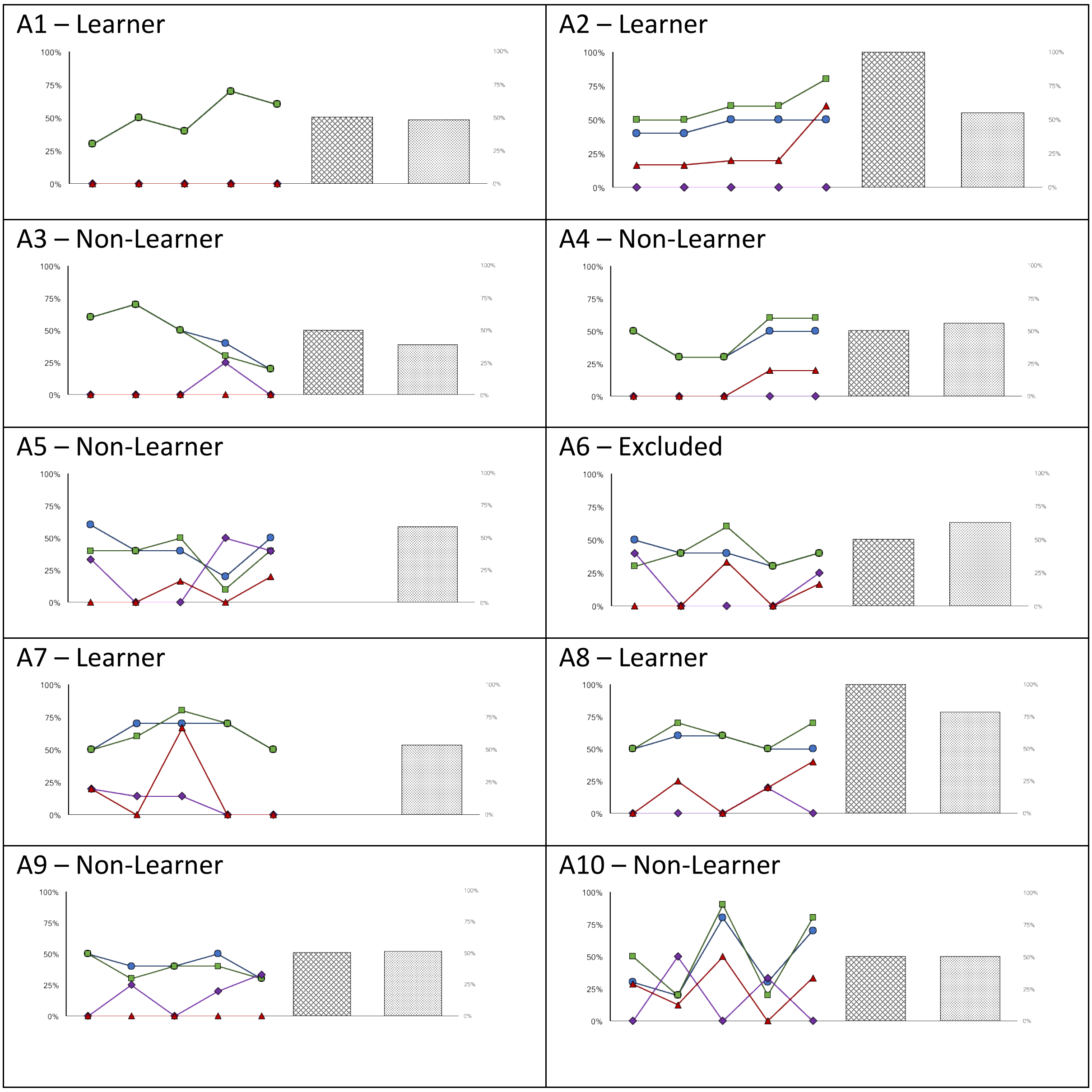

Supplementary Figure 1. Performance of bees trained on the ”above” protocol during training and transfer tests. Five blocks of 10 trials are presented with the percentage of correct choices. Green squares: number of correct feeders chosen, blue circles: selection of correct Y-maze arm first, red triangles: abandoned incorrect arm for a correct feeder (the higher the better), purple diamonds: abandoned correct arm for an incorrect feeder (the lower the better). Transfer test results, hatched bars: percentage of correct first touches, dotted bars: percentage of cumulative touches on correct feeder.

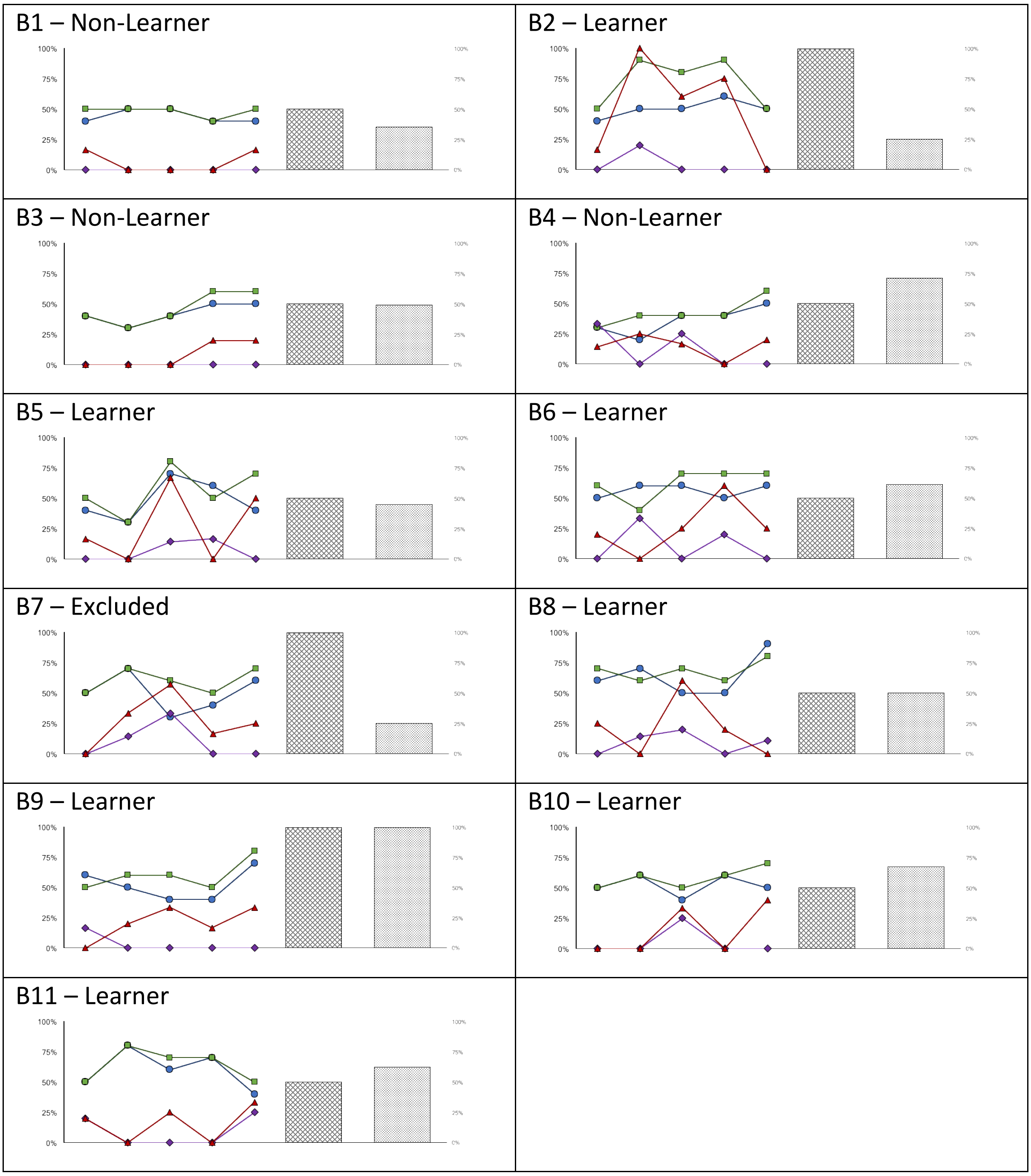

Supplementary Figure 2. Performance of bees trained on ‘below’ protocol during training and transfer tests. Five blocks of 10 trials are presented with the percentage of correct choices. Green squares: number of correct feeders, blue circles: selection of correct Y-maze arm first, red triangles: abandoned incorrect arm for a correct feeder (the higher the better), purple diamonds: abandoned correct arm for an incorrect feeder (the lower the better). Transfer test results, hatched bars: percentage of correct first touches, dotted bars: percentage of accumulative touches on correct feeder.

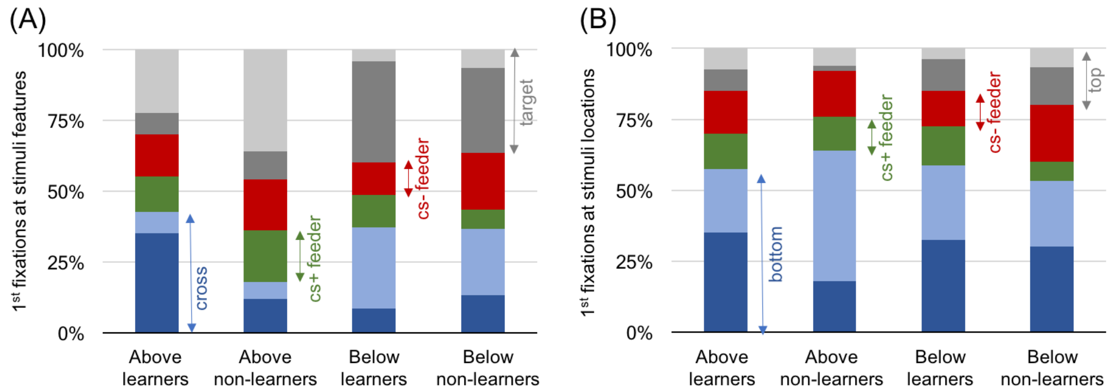

Supplementary Figure 3. Summary of learner and non-learner bees' initial fixation points over the last 10 training trials. (A) We analyzed which feature the bees inspected first. Blue: bee flew to referent cross shape first (dark blue: cross on rewarding stimuli (CS+), light blue: cross on incorrect punished stimuli (CS-)), green: bee flew directly to rewarding feeder, red: bee flew directly to CS- feeder, grey: bee flew to target shape first (dark grey: CS+ target, light grey: CS- target). (B) Analyses of the location that the bees flew to first. Blue: bee flew to the lowest shape on the stimuli first (dark blue: bottom shape on rewarding stimuli (CS+), light blue: bottom of incorrect stimuli (CS-)), green, red: direct flights to a feeder (as above), grey: bee flew to upper most shape on stimuli (dark grey: CS+ top, light grey: CS- top). An analysis of the 1st location point during the whole of training (per block of 10 trials) can be seen in Supplementary Figure 4.

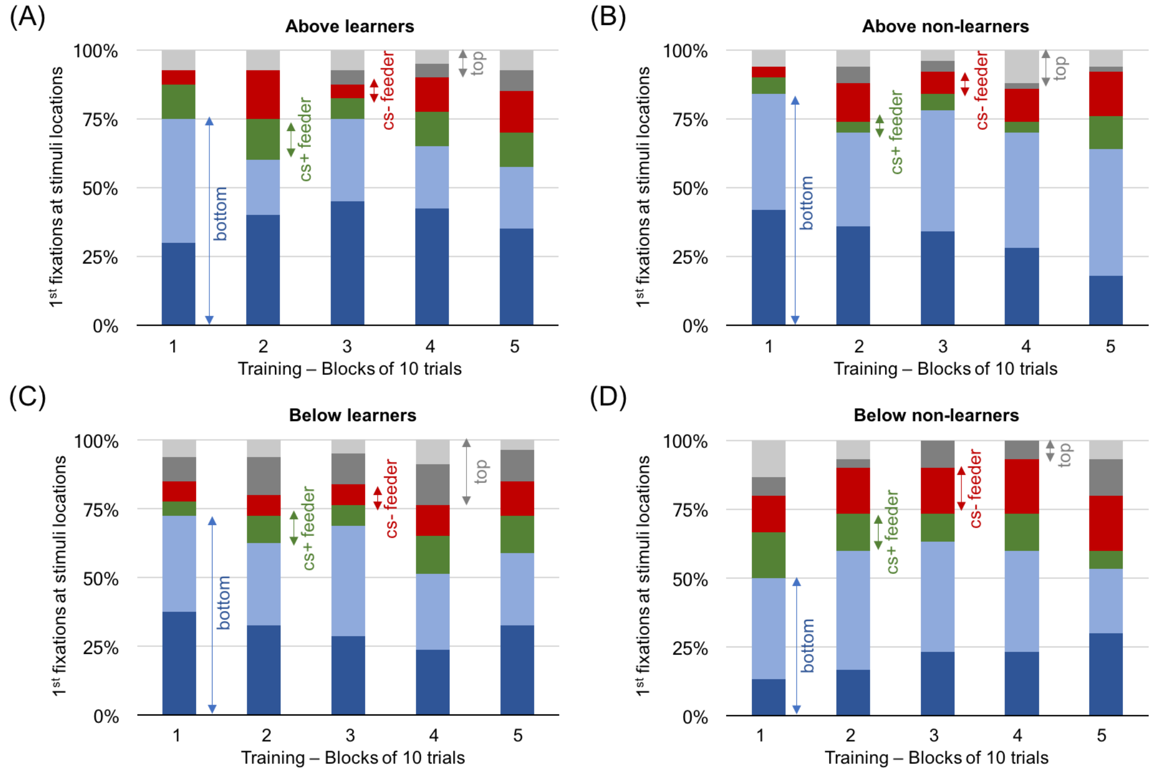

Supplementary Figure 4. Summary of learner and non-learner bees' initial fixation points over duration of training (blocks of 10 trials). Analyses of the location that the bees flew to first. Blue: bee flew to the lowest shape on the stimuli first (dark blue: bottom shape on rewarding stimuli (CS+), light blue: bottom of incorrect stimuli (CS-)), green, red: direct flights to a feeder (as above), grey: bee flew to upper most shape on stimuli (dark grey: CS+

top, light grey: CS- top). A) above protocol learner bees, B) above protocol non-learner bees, C) below protocol learner bees, D) below protocol non-learner bees,

Supplementary Table 1. Statistical results for training performances by block of 10 trials (Mann-Whitney U). Blue: results reported in the article. Bold: grouping for learners, non-learners and the test result for learners versus non-learners. A+: Above learners, A-: above non-learners, B+: below learners, B-: below non-learners; NS: non-significant.

**Training performances**	**Trials 1-10** ***X10***	**Trials 11-20** ***X20***	**Trials 21-30** ***X30***	**Trials 31-40** ***X40***	**Trials 41-50** ***X50***
**Learners vs chance (5)**	55.0; NS : 0.7414	33.0; 0.07672	**16.5; 0.00424**	**16.5; 0.00424**	**16.5; 0.00424**
**Non-learners vs chance**	24.0; NS : 0.42952	12.0; 0.04036	**20.0; NS : 0.22628**	**16.0; NS :0.1031**	**28.0; NS : 0.71138**
**A+ vs. B+**	**7.5; NS : 0.07463**	**12.5; NS : 0.42313**	**9.0; NS : 0.19089**	**14.0; NS : 0.46072**	**12.5; NS : 0.42183**
**A- vs B-**	**3.0; NS : 0.1004**	**5.5; NS : 0.32235**	**6.0; NS : 0.37697**	**3.5; NS : 0.14096**	**5.0; NS : 0.26932**
A+ vs A-	7.5; NS : 0.28025	3.5; NS : 0.06832	6.5; NS : 0.22838	1.5; 0.02407	5.0; NS : 0.13214
B+ vs B-	2.5; 0.02706	4.0; NS : 0.0818	0.5; 0.01346	3.0; NS : 0.05151	5.0; NS : 0.11984
A+ vs B-	4.0; NS : 0.27706	1.0; 0.04936	2.0; NS : 0.09737	1.5; NS : 0.07302	3.5; NS : 0.22936
A- vs B+	13.0; NS : 0.21921	7.5; NS : 0.05848	8.0; NS : 0.06875	3.0; 0.01079	8.0; NS : 0.0684
**Learners vs. Non-learners**	**32.0; NS : 0.13587**	**16.0; 0.01072**	**17.0; 0.01323**	**9.0; 0.00188**	**21.5; 0.03214**

Supplementary Table 2. Statistical results for tests at the end of the training paradigm (cumulative touches and 1st choice) per group. Blue: results reported in the article. Bold: grouping for learners, non-learners and the test result for learners versus non-learners. A+: Above learners, A-: above non-learners, B+: below learners, B-: below non-learners; NS: non-significative.

Groups	Test cumulative touches	Test 1st choice
**Learners vs. Chance**	**22.0; 0.0**	**38.5; 0.045**
**Non-learners vs. chance**	**16.0; 0.173**	**28.0; 0.335**
**A+ vs. B+**	**55.5; NS : 0.5**	**55.0; NS : 0.48368**
**A- vs B-**	**28.5; NS : 0.45675**	**27.0; NS : 0.37639**
A+ vs A-	32.0; NS : 0.25181	31.0; NS : 0.19195
B+ vs B-	33.5; NS : 0.25348	36.0; NS : 0.29669
A+ vs B-	14.5; NS :	21.0; NS :

	0.1216	0.35362
A- vs B+	57.5; NS : 0.24004	53.0; NS : 0.13167
**Learners vs. Non-learners**	137.5; NS : 0.12958	30.0; NS : 0.069

Supplementary Table 3. Statistical results for the choice of the correct arm from decision chamber tested versus chance (2nd column) and the difference when the bee is doing a correct choice of feeder after a correct choice of arm versus the correct choice of arm (no difference). In blue the results reported in the article. Bold: grouping for learners, non-learners and the test result for learners versus non-learners. A+: Above learners, A-: above non-learners, B+: below learners, B-: below non-learners; NS: non-significant.

Groups tested: ***Correct arm vs. chance***	*Mann-Whitney U ; p-value*	*Groups tested:* ***CACC vs. CA***
**Learners vs. chance**	**33.0; 0.07672**	**42.0; NS : 0.238**
**Non-learners vs. chance**	**0.0; 0.00094**	**23.0; NS : 0.37346**
**All bees vs chance**	152.0; NS : 0.41222	147.0; NS : 0.16614
**A+ vs. B+**	12.0; NS : 0.38766	
**A- vs B-**	5.5; NS : 0.32235	
A+ vs A-	1.5; 0.02407	
B+ vs B-	0.5; 0.01468	
A+ vs B-	1.0; NS : 0.05581	
A- vs B+	0.5; 0.00353	
**Learners vs. Non-learners**	**3.5; 0.00045**	

Supplementary Table 4. Results of Mann-Whitney U tests for choice of the incorrect arm as first choice. Each group is tested against chance (50/2 = 25), compared with the choices for the correct and incorrect arm. Blue: results reported in the article. Bold: grouping for learners, non-learners and the test result for learners versus non-learners. A+: Above learners, A-: above non-learners, B+: below learners, B-: below non-learners; NS: non-significant.

Groups tested: **Incorrect arm vs. chance**	Mann-Whitney U ; p-value<br><br>IC vs chance	Groups tested : **ICCC vs. IC**
**L vs. chance**	33.0; 0.07672	0.0; 0.00008
**NL vs. chance**	0.0; 0.00094	0.0; 0.00094
All bees	152.0; NS : 0.18478	0.0; 0.0
**A+ vs. B+**	**12.0; NS : 0.38766**	
**A- vs B-**	**3.5; NS : 0.13312**	
A+ vs A-	0.5; 0.01216	
B+ vs B-	0.5; 0.01468	
A+ vs B-	1.0; NS : 0.05581	
A- vs B+	0.0; 0.00267	
**Learners vs. Non-learners**	**2.0; 0.00028**	

Supplementary Table 5. Results of Mann-Whitney U tests for 1st choice scanning behavior (bottom or top item, feeders). Each group is tested against each other and against chance (50/3 = 16.67). In blue the results reported in the article. Bold: grouping for learners, non-learners and the test result for learners versus non-learners. A+: Above learners, A-: above non-learners, B+: below learners, B-: below non-learners; NS: non-significant. The three important positions where the bee can perform scan behaviors are the bottom item of the sheet (bottom), the top item (top) or the feeder.

1st item scanned		*Mann-Whitney U, p-value*	*Mann-Whitney U, p-value*	*Mann-Whitney U, p-value*		*Bottom item*	*Feeders*	*Top item*
		bottom vs top	***bottom vs feeder***	***top vs feeders***		***Vs chance***	***Vs chance***	***Vs chance***
**Learners**		0.0; 4e-05	0.0; 4e-05	28.0; 0.01708	L vs chance	0.0; 1e-05	22.0; 0.00379	11.0; 0.00029
**Non-learners**		0.0; 0.00045	0.0; 0.00046	6.0; 0.00359	NL vs chance	0.0; 0.0002	0.0; 0.0002	0.0; 0.0002
**All bees**		0.0; 0.0	0.0; 0.0	70.5; 0.00067	**All bees vs. chance**	**0.0; 0.0**	38.0; 0.0	19.0; 0.0
		1st choice = bottom item	***1st choice = feeders***	***1st choice = top item***				
**A+ vs B+**		**13.0; NS : 0.46184**	**10.0; NS : 0.25222**	**8.5; NS : 0.16901**				
**A- vs B-**		**4.0; NS : 0.18266**	**1.0; 0.03682**	**7.0; NS : 0.5**				
A+ vs A-		7.0; NS : 0.26026	8.5; NS : 0.40285	8.0; NS : 0.35608				
B+ vs B-		7.0; NS : 0.24706	3.0; NS : 0.05477	10.0; NS : 0.5				
A+ vs B-		3.0; NS : 0.18623	3.0; NS : 0.18838	5.0; NS : 0.42921				
A- vs B+		17.0; NS : 0.5	9.0; NS : 0.09577	16.5; NS : 0.46736				
**Learners vs. Non-learners**		**34.0; NS : 0.21373**	**23.5; 0.04881**	**41.5; NS : 0.43407**				

Supplementary Table 6. Results of Mann-Whitney U tests for 2nd choice scanning behavior (bottom or top item, feeders). Each group is tested against each other and against chance (50/3 = 16.67). In blue the results reported in the article. In bold, the grouping for learners, non-learners and the test result for learners versus non-learners. A+: Above learners, A-: above non-learners, B+: below learners, B-: below non-learners; NS: non-significant. The three important positions where the bee can perform scan behaviors are the bottom item of the sheet (bottom), the top item (top) or the feeder.

2nd item scanned	*N*	*Mann-Whitney U, p-value*	*Mann-Whitney U, p-value*	*Mann-Whitney U, p-value*		*Bottom item*	*Feeders*	*Top item*
		bottom vs top	***bottom vs feeder***	***top vs feeders***		***Vs chance***	***Vs chance***	***Vs chance***
**Learners**		51.0; NS : 0.27517	0.0; 4e-05	0.0; 4e-05	L vs chance	11.0; 0.00028	0.0; 1e-05	0.0; 1e-05
**Non-learners**		26.5; NS : 0.29881	0.0; 0.00046	0.0; 0.00046	NL vs chance	0.0; 0.0002	0.0; 0.0002	0.0; 0.0002
**All bees**		147.0; NS : 0.16583	0.0; 0.0	0.0; 0.0	**All bees vs chance**	19.0; 0.0	**0.0; 0.0**	0.0; 0.0
		2nd choice = bottom item	***2nd choice = feeders***	***2nd choice = top item***				
**A+ vs. B+**		**11.0; NS : 0.31352**	**11.5; NS : 0.35241**	**13.5; NS : 0.5**				
**A- vs B-**		**4.5; NS : 0.22668**	**6.5; NS : 0.4404**	**5.0; NS : 0.27308**				
A+ vs A-		6.5; NS : 0.22643	6.5; NS : 0.23028	9.0; NS : 0.44956				
B+ vs B-		10.0; NS : 0.5	7.0; NS : 0.24706	9.0; NS : 0.40958				
A+ vs B-		3.5; NS : 0.23574	6.0; NS : 0.42984	4.5; NS : 0.36064				
A- vs B+		9.5; NS : 0.10751	7.0; NS : 0.05158	13.0; NS : 0.25574				
**Learners vs. Non-learners**		**35.5; NS : 0.25054**	**26.5; NS : 0.07974**	**37.5; NS : 0.30891**				

Supplementary Table 7. Number of scanning behaviors displayed during training and when making correct or incorrect choices. Blue: results reported in the article. Bold: grouping for learners, non-learners and the test result for learners versus non-learners. A+: Above learners, A-: above non-learners, B+: below learners, B-: below non-learners; NS: non-significant.

Variables : total correct choices	*Test (N, Mann-Whitney U, p-value)*	*Test (N, Mann-Whitney U, p-value)*	*Test (N, Mann-Whitney U, p-value)*
Groups tested	*Number of scanning behaviour during training (NS)*	***NS when correct choices***	***NS when incorrect choices***
**A+ vs. B+**	48.0; NS : 0.29219	5.0; NS : 0.0541	11.0; NS : 0.31792
**A- vs B-**	27.5; NS : 0.40838	3.0; NS : 0.11652	3.0; NS : 0.11652
A+ vs A-	33.0; NS : 0.26861	3.0; NS : 0.05567	1.0; 0.01827
B+ vs B-	40.5; NS : 0.46489	7.0; NS : 0.24706	9.0; NS : 0.40985
A+ vs B-	21.0; NS : 0.36518	6.0; NS : 0.42984	5.0; NS : 0.42921
A- vs B+	69.5; NS : 0.5	4.0; 0.01738	8.0; NS : 0.07193
**Learners vs. Non-learners**	**167.0; NS : 0.39411**	**20.0; 0.02616**	**28.0; NS : 0.1002**
